# “Send My Information”: Increasing public accessibility to clinical trials by facilitating participant expression of interest

**DOI:** 10.1017/cts.2022.19

**Published:** 2022-02-11

**Authors:** Leah Dunkel, Loretta M. Byrne, Erik Olson, Michael Russell, Jason Tan, Kaysi Phillips, Consuelo H. Wilkins, Paul A. Harris

**Affiliations:** 1 Vanderbilt Institute for Clinical and Translational Research, Vanderbilt University Medical Center, Nashville, TN, USA; 2 Department of Medicine, Vanderbilt University Medical Center, Nashville, TN, USA; 3 Department of Internal Medicine, Meharry Medical College, Nashville, TN, USA; 4 Office of Health Equity, Vanderbilt University Medical Center, Nashville, TN, USA; 5 Department of Biomedical Informatics, Vanderbilt University Medical Center, Nashville, TN, USA

**Keywords:** Trial enrollment, clinical trials, research recruitment, research infrastructure, patient information needs, willingness to participate

## Abstract

**Introduction:**

The process of identifying and connecting with clinical trial study teams can be challenging and difficult for members of the public. The national volunteer community registry, ResearchMatch, and the public clinical trials search tool, Trials Today, work in tandem to bridge this connection by providing a streamlined process for potential participants to identify clinical trials which may be of interest.

**Methods:**

Building on the existing infrastructure of ResearchMatch and Trials Today, we created a mechanism by which the public can request that their basic contact information (e.g., email/phone) be securely shared with any actively recruiting clinical trial, including trials with no existing relationship with ResearchMatch.

**Results:**

Within the first 2 years of use (July 2019–July 2021), ResearchMatch Volunteers sent 12,251 requests to study teams. On average, 20% of these requests were accepted by the study teams.

**Conclusions:**

The utilization of this tool indicates that there is active interest among members of the public to independently contact study teams about trials of interest. Additionally, research teams unaffiliated with ResearchMatch are willing to at minimum accept contact information. This allows ResearchMatch to successfully serve as a medium, connecting members of the public with actively recruiting trials.

## Introduction

Despite efforts made by clinical researchers, clinical trials often fail to meet recruitment goals or need extended trial timelines to reach total accrual [1]. It has been shown that several factors play a role in preventing physicians from sharing clinical trial information with patients and that patients are not sure how to find trial information themselves [2–4]. Attempts by patients to independently find clinical trial information using online resources has been described as cumbersome and difficult [5,6]. If an individual is able to locate a trial which may meet their objectives, contacting the research team is often the next challenge. They may be intimidated, reticent, or reluctant to directly initiate the first contact with unknown investigators. These challenges faced by research teams and patients are highlighted in the Institute of Medicine’s (IOM) agenda for 2020 [7]. The IOM calls for novel approaches to facilitate connectivity between these two clinical trial enterprise stakeholder groups.

To that end ResearchMatch, (www.researchmatch.org) [8] a disease and institution neutral national recruitment registry project unites these two groups to facilitate enrollment of participants for clinical trials. ResearchMatch currently supports over 154,000 self-registered members who are referred to as “Volunteers,” as well as 10,000 researchers from 186 leading research institutions in the USA. The ResearchMatch platform connects or “matches” researchers with registered Volunteers, at no cost, via an interactive email clearinghouse model initiated by the researcher.

Trials Today at ResearchMatch [9] was launched in 2015 as a disease-neutral clinical trial search engine to promote public awareness of recruiting clinical trials and as a mechanism allowing ResearchMatch Volunteers and members of the public to take a more active role in self-identification of relevant clinical trials. Trials Today utilizes the Unified Medical Language System, created by the National Institutes of Health (NIH) National Library of Medicine to provide semantic matching of self-reported medical conditions of interest. Any clinical trial currently listed as “recruiting” on ClinicalTrials.gov is listed and searchable. Trials Today offers a public-friendly entry point to the more cumbersome clinical trial search engine ClinicalTrials.gov which was designed for regulatory reporting rather than lay public interaction.

While individuals using trial identification tools (e.g., ClinicalTrials.gov, Trials Today) may find a pertinent study for their health condition, the onus of making first contact with the study investigator can be intimidating and may result in oversharing information (e.g., Volunteer sending protected health information via email that is unrelated to the study).The bidirectional connectivity model we describe here, the “Send My Information” (SMI) feature, establishes a novel and reliable means by which an individual may initiate the first interaction with a trial investigator in a secure and guarded manner facilitated by ResearchMatch and Trials Today. This paper provides detail on the methods used to create SMI and its utilization over the first 2 years. We describe how the public users’ personal information is secured thereby providing a level of information protection over simple email connectivity. We also present lessons learned that should be of use to other teams attempting to increase public awareness and public participation in biomedical research.

## Methods

### Integration of Trials Today with ResearchMatch

Beginning in 2018, clinical trial listings pulled from Trials Today, and relevant to their self-reported health conditions, were embedded and displayed on the individual ResearchMatch Volunteers dashboard. These listings are accessible to the Volunteer once logged in and correspond to the Volunteer’s reported health conditions, age, gender, and self-reported willingness to travel to participate in a study.

Given positive feedback that ResearchMatch Volunteers were interested in self-initiated methods for trial identification, we focused next on a streamlined model for making initial contact with trial recruiters. The SMI tool creates a seamless infrastructure by which Volunteers may search for and initiate contact with any actively recruiting clinical trial (regardless of that trial’s relationship with ResearchMatch) that interests them and be contacted in return for follow-up questions and consideration. This approach encourages Volunteers to express interest in studies which may not be registered on the ResearchMatch platform such as industry sponsored trials occurring at nonacademic research institutions.

For individuals interested in using the SMI tool who were not already ResearchMatch Volunteers, we designed the SMI workflow to guide them through the ResearchMatch sign-up process. Joining ResearchMatch as a Volunteer is required only once and allows unlimited Volunteer-driven SMI contact engagements along with opportunities for passive receipt of study opportunities from ResearchMatch research teams.

### Stakeholder and Community Input

Prior to development, we engaged our local Institutional Review Board (IRB), Privacy Office, and the ResearchMatch Governance Team. Within the SMI model, ResearchMatch extends into previously uncharted waters by advocating on behalf of the Volunteer who seeks to share their contact information with study teams that have no prior affiliation with ResearchMatch, all while maintaining Volunteer privacy and security. Early stakeholder engagement meetings stressed that Volunteers must be presented with clear descriptions of the process as well as multiple opportunities to confirm their desire to share their contact information.

We sought to ensure that members of the lay public contributed to and informed the evolution of this tool from initial development to release (see Table 1). The SMI process was reviewed on multiple occasions by the Recruitment Innovation Center Community Advisory Board (RIC CAB). The RIC CAB includes a diverse, national representation from community members, patient advocates, nonprofit leaders, and community physicians [10]. In addition to providing feedback on the overall SMI workflow, the RIC CAB recommended ensuring Volunteers are informed throughout the SMI process and that educational resources (e.g., email templates for responding to Volunteers) be provided to research teams.

**Table 1.** Stakeholder feedback and solutions

Stakeholder feedback and design modifications		
Stakeholder	Advice	Design solution
Community Members	Offer choice as to what information shared Alert when information is accepted or rejected	Volunteer chooses what contact information to share Volunteer notified by email when trial contact receives information.
IRB and Privacy Office	Ensure Volunteer information is protected	Requests can only be accessed for 14 days. Only one individual may accept contact information.
ResearchMatch Governance Team	Establish legitimacy of ResearchMatch for study teams not familiar with the platform	NIH funding noted in email with links to official ResearchMatch page

To explore acceptance from the larger research community and that there would be an interest and receptiveness from clinical trial recruiters with no prior affiliation with ResearchMatch or Trials Today, we contacted a small sample of studies listed on clinicaltrials.gov and inquired if they would theoretically accept contact information sent by ResearchMatch. Approximately half of those emailed replied favorably, while the remaining half did not respond to the email inquiry.

### How Send My Information Works

**Fig. 1:**
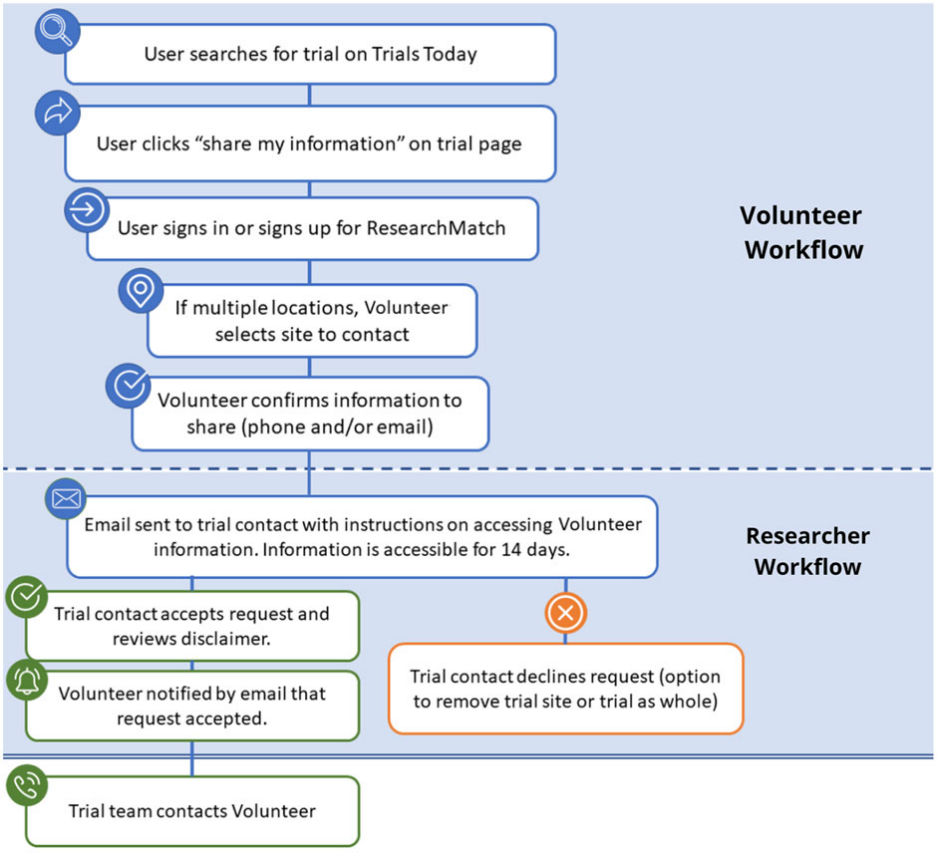
Send My Information (SMI) workflow.

The SMI workflow was designed to seamlessly fit into the existing Trials Today interface. After identifying a trial of interest, the user begins the SMI process by clicking a SMI button that is prominently listed by each displayed trial opportunity. The user then makes a series of selections detailing what information will be shared and with whom (Fig. 1). If a trial includes multiple recruiting sites, the sites are listed by distance, and the user has the ability to choose the site they wish to contact.

After an SMI request is submitted, the contact listed for the trial (trial contact) receives an email indicating that a Volunteer is interested in the clinical trial and seeks to share their contact information (email, phone number, or both). This request must be accepted within 14 days. To accept the potential Volunteer’s contact information, the trial contact must complete the following required steps:

1. Review the terms of accepting the Volunteer’s contact information, including agreement that connection is only applicable to the referenced trial;
2. Enter the name and organizational affiliation of the individual accepting the information;
3. The Volunteer’s contact information is then displayed for a period of 5 min, allowing the trial contact to copy the information and subsequently reach out to the Volunteer directly. This information will only be displayed once and, after the 5 min window, the information cannot be accessed again.

After the trial contact completes this process, ResearchMatch notifies the Volunteer that their contact information was accepted.

Recognizing that not all trial teams would be receptive to “cold call” contacts by Volunteers through the ResearchMatch SMI workflow, we created a simple feedback option for trial teams to opt out of future contact requests. After receiving a contact request, study team members may indicate that they would like to no longer receive requests for either (1) an individual trial recruitment site or (2) the entire trial (if the site serves as the coordinating center).

### Design Specifications

Protection of identifiable Volunteer information is paramount, both for required compliance and to ensure continued trust within the ResearchMatch Volunteer community. Therefore, we implemented safeguards controlling who could access the information and for what length of time.

Access to the Volunteer’s information is allowed only once to minimize risk of contact information being shared. To accommodate variation and autonomy of trial teams, the trial contact may forward the still unaccepted share request (i.e., forward email) to the appropriate site. The appropriate site contact would then access the link and accept the Volunteer’s information by entering their own name and contact information.

**Fig. 2:**
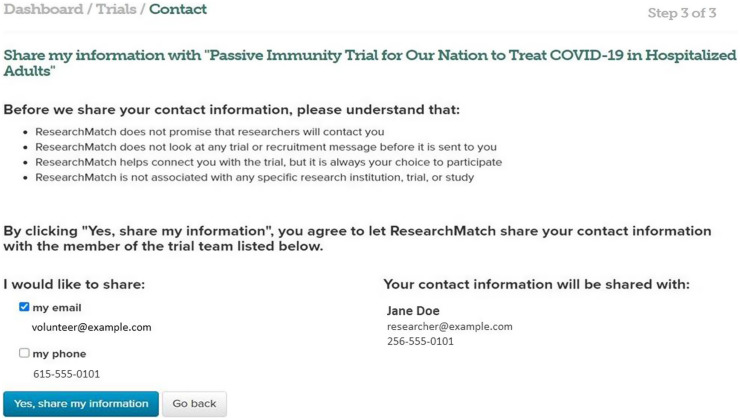
Send My Information final confirmation page.

Prior to sending an SMI request, Volunteers are asked to confirm preferred contact information that they would like to send and are notified once their information is shared with the study team (see Fig. 2).

After accepting Volunteer contact information, research teams are advised to follow up with the Volunteer in a timely manner and to alert the Volunteer that their information was received through SMI (e.g., “I received your contact message via Trials Today). Ideally, rapid follow-up will improve the potential to establish rapport and trust between study teams and the Volunteer.

### Data Logging

ResearchMatch maintains a record of each SMI request made as well as the request outcome. In this way, we may ascertain which trials receive the most contacts and how many of those requests are accepted and aggregate demographic metrics of Volunteers using the tool. We used descriptive statistics (e.g., counts, proportions, and means) to characterize the SMI experience to date.

## Results

### Usage

**Fig. 3:**
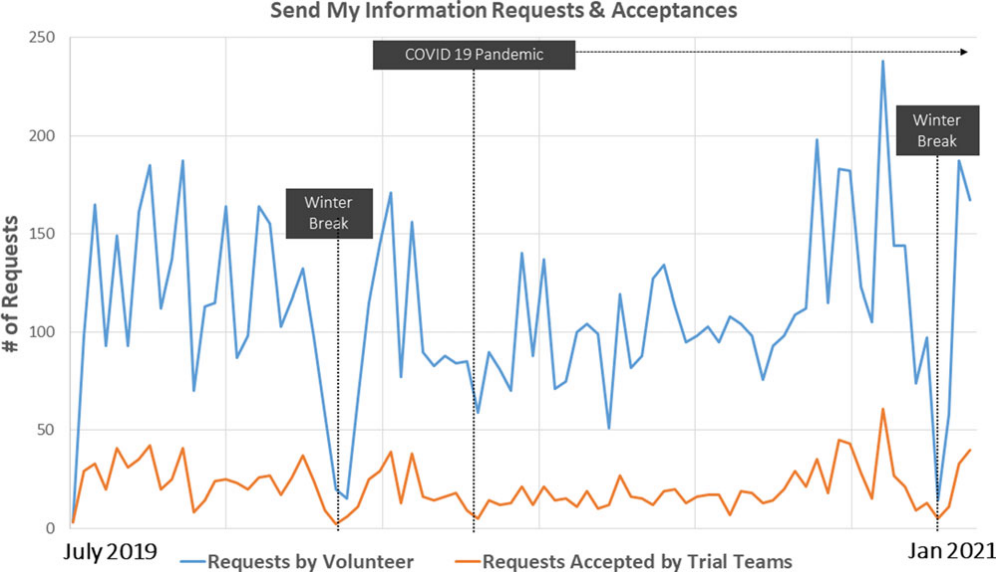
Send requests and acceptances by study teams.

The SMI workflow launched in July 2019 and has seen consistent use by the ResearchMatch Volunteer community (Fig. 3) with an average of approximately 117 requests sent each week. This number tends to decrease over winter holidays, along with the acceptance rate, perhaps due to temporary closing of institutions when research teams may be out of the office. Throughout the initial 2 years of operating of the SMI model (July 2019–July 2021), weekly acceptance rates have ranged from 6% to 33%, with an average acceptance of 20%. Notably, neither use of SMI nor the acceptance rate drastically changed throughout the COVID-19 pandemic.

**Fig. 4:**
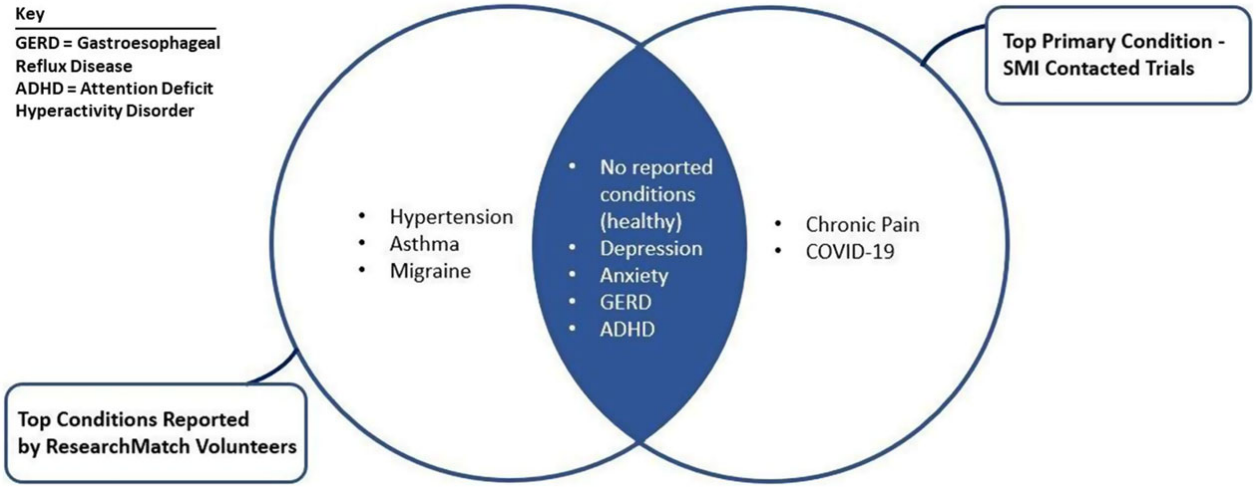
Top reported conditions on ResearchMatch and primary condition of contacted trials. SMI, Send My Information.

Trials with the most interest, as reflected by the largest number of SMI requests sent, focus on mental health (e.g., depression and anxiety) and chronic pain. Of note, this overlaps significantly with top reported conditions among the ResearchMatch Volunteer community, which includes depression, anxiety, and chronic pain (see Fig. 4).

### User Description

From July 2019 to July 2021, over 12,000 SMI requests were sent by 4108 unique Volunteer users. This indicates an average of slightly less than three requests per Volunteer opting to use the SMI service. As of July 2021, the ResearchMatch community included 154,906 Volunteers. As all SMI users are either existing Volunteers or new Volunteers who sign up in order to send an SMI request, we can estimate that 2% of the Volunteer base sent at least one SMI request. Overall, the demographics of those using the SMI tool largely mirror the larger ResearchMatch Volunteer base – predominantly female, White, and non-Hispanic (see Table 2) [11].

**Table 2.** User characteristics

User demographics		
Category	SMI users	ResearchMatch volunteers
Gender		
Female	71.4% (2746)	70.4%
Male	27% (1043)	29%
Transgender	1.4% (55)	0.6%
Race		
American Indian or Alaska Native	1% (39)	0.8%
Asian	3.3% (130)	3.8%
Black or African American	8.2% (316)	11.3%
Multi-Racial	6% (236)	5.2%
Native Hawaiian or Other Pacific Islander	>1% (5)	>1%
Other	2.6% (101)	3.1%
White	78.4% (3017)	75.5%
Ethnicity		
Hispanic or Latino	9.6% (368)	8.9%
Not Hispanic or Latino	90.4% (3476)	91.1%
Total	4108	155,202

Sum of total Send My Information (SMI) users differs from total unique users as some Volunteers have since deleted their account and demographics are no longer accessible.

Notably, use does include individuals from all race and ethnicity groups and represents individuals living across all 50 states and the District of Columbia. The largest number of requests came from individuals living in California and Ohio, which again mirrors the existing ResearchMatch Volunteer population base [11].

While we did not actively capture the age of individuals using the SMI tool, we would expect this to also mirror the larger ResearchMatch community, which is approximately 4% aged <18 years, 33% aged 18–29 years, 38% aged 30–49 years, 21% aged 50–70 years, and 4% aged 70+ years.

### Impact

During the first 2 years of use (July 2019–July 2021), the SMI tool provided 2399 successful initial connections (i.e., accepted contact information) between members of the public and research teams.

Of all requests, 5774 (47%) were sent to study contacts with email addresses ending in “.edu,” indicating an academic affiliation. In addition, 1296 (11%) requests were sent directly to NIH-affiliated trials. Of trials contacted using the SMI mechanism whose study team accepted contact information, 15% were registered with ResearchMatch, indicating that the tool successfully facilitates connections regardless of prior ResearchMatch affiliation.

Top contacted trials (Table 3) focused on therapies for treatment-resistant depression, other mental health topics, and COVID-19.

**Table 3.** Top 5 contacted trials

NCTID	Study title	Requests sent	ResearchMatch Registration
NCT03775200	The Safety and Efficacy of Psilocybin in Participants With Treatment Resistant Depression (P-TRD)	306	Registered with ResearchMatch
NCT03944447	Outcomes Mandate National Integration with Cannabis as Medicine for Prevention and Treatment of COVID-19 (OMNI-Can)	159	No ResearchMatch Registration
NCT04377100	Impact on Anxiety and Motivation of COVID-19 and Predictors of Individual Responses	82	Registered with ResearchMatch
NCT03866174	A Study of Psilocybin for Major Depressive Disorder (MDD)	52	No ResearchMatch Registration
NCT04516746	Phase III Double-blind, Placebo-controlled Study of AZD1222 for the Prevention of COVID-19 in Adults	48	No ResearchMatch Registration

#### Registration with ResearchMatch

While not the primary goal of the SMI mechanism, we saw an increase in the number of Volunteers registered with ResearchMatch. From launch to July 2021, 661 new Volunteers joined ResearchMatch via the SMI sign-up mechanism.

#### Researcher Request for Removal

To date (July 2021), 135 study teams out of 3969 contacted opted out of future contact. Reasons for refusal largely consisted of study closure and completed recruitment. Some studies indicated that recruitment was on hold due to the COVID-19 pandemic. One study was only recruiting for international sites and thus removed themselves from further SMI contact. The SMI option was removed for studies which responded to SMI requests with an automated reply given this is an indicator that mailboxes are not actively monitored.

## Discussion

The results from the first 2 years indicate that members of the lay public have an active and vested interest in directly contacting research teams and participating in clinical trials. The SMI tool in ResearchMatch provides an easy and clear way for the lay public to independently indicate interest in studies. While more education and outreach are needed to increase the acceptance rate by study personnel, many study teams are willing to accept information shared by ResearchMatch even without a preexisting familiarity with the platform.

### Lessons Learned

As with any novel tool, there are opportunities to improve the use and functionality, largely by providing additional direction and detail for study teams during initial contact.

For some study teams, there is an understandable hesitancy to engage with ResearchMatch, an entity with which they may have no prior experience and limited ability to verify. After receiving a contact request, several research teams indicated to our team that they “could not accept contact information in this manner.” Adherence to strict protocol guidelines is of paramount concern to study teams and these teams may be concerned that accepting the information would qualify as a protocol noncompliance. In most cases, these concerns were resolved through direct communication with the study team. The SMI tool is, at its core, no different than an email or phone call from an interested member of the public. Other teams requested that the Volunteer contact the site directly to learn more about the study, which renders the SMI tool ineffective.

It became clear through feedback received by study teams that more information was required in the email sent to the trial contact. Coordinating centers requested that the email include the study site selected by the Volunteer to allow forwarding of the email request to the appropriate site. In response, both the specific site selected by the Volunteer and the full study title were added to the email sent to study teams.

### Limitations

There are four key barriers to connecting the Volunteer with the study team:

#### ResearchMatch Familiarity

Trials Today supports over 22,000 research trials and many are not registered on ResearchMatch. The research teams on these trials potentially contacted by ResearchMatch may be unfamiliar with or have limited awareness of ResearchMatch. Consequently, we suspect that some study teams may simply dismiss the contact request process email.

#### IRB Considerations

Some trial teams reported to be limited by their IRB-approved protocol, in how participants can be identified, and thus did not feel comfortable accepting contact information. In the near future, we will include downloadable information about ResearchMatch and the SMI process in the communications to study teams. These materials may be shared with concerned IRBs to alleviate concerns about obligations and the mechanisms of the exchanged information.

#### Technology Access

The SMI process is initiated by members of the lay public including patients. To use the tool, users must be an existing ResearchMatch Volunteer or agree to register on ResearchMatch, which requires an active email address. While SMI allows the Volunteer to indicate that they prefer to be contacted by phone, those with limited Internet and/or email access may still be unable to register and use this feature.

#### Health Literacy

Many trial descriptions are written in medical terms and may lack cultural considerations as well. Despite efforts to organize information into readable, comprehensive sections on Trials Today, we are not able to control the wording used in trial descriptions. The issues faced by people with lower health literacy have been shown to result in lower rates of use of clinical trial search engines [12].

#### Evaluation

The SMI tool is currently limited in terms of ability to measure and evaluate trial enrollment. While we measure the number of “matches” made between research teams and interested Volunteers, this does not equate to rigorous screening or enrollment in the trial itself. Tracking Volunteers and teams after initial connection is beyond the scope of this SMI workflow project, but an important consideration in future development work.

### Future Development

For further iterations of SMI and other connectivity methods like it, providing clarity on the source of information, the mechanism of the origin, and any associated obligations on the part of the stakeholders is critical. We plan to continue efforts to increase awareness and familiarity among research teams and institutions with regard to both the SMI tool and ResearchMatch more broadly.

To increase transparency for Volunteers, we hope to build individual dashboards listing the trials contacted and the status of each request (e.g., accepted, not accepted, and expired). In this way, Volunteers can clearly see the outcome of each request.

Beyond process improvements and increasing understanding among Volunteers and research teams, next step planning for SMI includes additional effectiveness evaluation of this contact mechanism. This may involve a pilot study asking both Volunteers and research teams to provide feedback on the outcome of the contact request, as well as developing other strategies to track these requests beyond the initial point of contact and measure the extent to which the SMI connectivity results in later enrollment.

### Conclusions

The SMI feature demonstrates that there is willingness among the lay public to directly contact trials when they are assisted by ResearchMatch. There is a considerable percentage (approximately 20%) of trial teams who will accept the contact information of these individuals. This novel tool successfully provides a means for the public to make first contact when a safeguard is applied by ResearchMatch. Future work will focus on evaluating experiences of both research teams and Volunteers beyond the initial contact and exploring potential to highlight studies of national importance.
